# Texture Analysis Improves the Value of Pretreatment ^18^F-FDG PET/CT in Predicting Interim Response of Primary Gastrointestinal Diffuse Large B-Cell Lymphoma

**DOI:** 10.1155/2020/2981585

**Published:** 2020-08-21

**Authors:** Yiwen Sun, Xiangmei Qiao, Chong Jiang, Song Liu, Zhengyang Zhou

**Affiliations:** ^1^Department of Nuclear Medicine, Nanjing Drum Tower Hospital Clinical College of Nanjing Medical University, Nanjing 210008, China; ^2^Department of Radiology, Nanjing Drum Tower Hospital Clinical College of Nanjing Medical University, Nanjing 210008, China

## Abstract

**Objectives:**

To explore the application of pretreatment ^18^F-fluorodeoxyglucose (^18^F-FDG) positron emission tomography (PET)/computed tomography (CT) texture analysis (TA) in predicting the interim response of primary gastrointestinal diffuse large B-cell lymphoma (PGIL-DLBCL).

**Methods:**

Pretreatment ^18^F-FDG PET/CT images of 30 PGIL-DLBCL patients were studied retrospectively. The interim response was evaluated after 3-4 cycles of chemotherapy. The complete response (CR) rates in patients with different clinicopathological characteristics were compared by Fisher's exact test. The differences in the maximum standard uptake value (SUVmax), metabolic tumor volume (MTV), and texture features between the CR and non-CR groups were compared by the Mann–Whitney *U* test. Feature selection was performed according to the results of the Mann–Whitney *U* test and feature categories. The predictive efficacies of the SUVmax, MTV, and the selected texture features were assessed by receiver operating characteristic (ROC) analysis. A prediction probability was generated by binary logistic regression analysis.

**Results:**

The SUVmax, MTV, some first-order texture features, volume, and entropy were significantly higher in the non-CR group. The energy was significantly lower in the non-CR group. The SUVmax, volume, and entropy were excellent predictors of the interim response, and the areas under the curves (AUCs) were 0.850, 0.805, and 0.800, respectively. The CR rate was significantly lower in patients with intestinal involvement. The prediction probability generated from the combination of the SUVmax, entropy, volume, and intestinal involvement had a higher AUC (0.915) than all single parameters.

**Conclusions:**

TA has potential in improving the value of pretreatment PET/CT in predicting the interim response of PGIL-DLBCL. However, prospective studies with large sample sizes and validation analyses are needed to confirm the current results.

## 1. Introduction

The incidence of non-Hodgkin's lymphoma (NHL), especially extranodal lymphoma, has increased during the last several decades [1, 2]. The gastrointestinal (GI) tract is the extranodal site most frequently involved in NHL [3]. The histopathological subtypes of primary gastrointestinal lymphoma (PGIL) are diverse. Diffuse large B-cell lymphoma (DLBCL) is the most common subtype of PGIL. The clinical-histologic feature, response, and outcome of patients with PGIL-DLBCL have been reported to be different from those with nodal original DLBCL [1]. Thus, it is important to study PGIL-DLBCL as a special cohort.

The importance of personalized precision medicine has been highlighted recently. In PGIL-DLBCL, considering the postoperative complications of traditional surgical resection [3] and the development of chemotherapeutics, especially the emergence of rituximab, R–CHOP regimen chemotherapy (rituximab, cyclophosphamide, doxorubicin, vincristine, and prednisone) has replaced surgical treatment to become the first-line therapy [4]. Despite advances of the R–CHOP regimen, it has been reported that approximately 40% of DLBCL patients are not sufficiently cured [5] and may need alternative therapies such as salvage regimen, transplantation, radiation therapy, and chimeric antigen receptor (CAR) T-cell therapy [6–8]. Thus, pretreatment prediction of treatment response and prognosis is important in disease management.

The use of ^18^F-fluorodeoxyglucose (^18^F-FDG) positron emission tomography (PET)/computed tomography (CT) in Hodgkin's lymphoma (HL) and aggressive NHL has been widely approved [9]. Although the use of ^18^F-FDG PET/CT in some subtypes of PGIL is controversial (such as mucosa-associated lymphoid tissue lymphoma, which was reported to have a lower detection rate and FDG-avidity [10]), it plays an important role in staging, treatment response evaluation, and follow-up in DLBCL. As semiquantitative interpretation methods of ^18^F-FDG PET/CT, the standard uptake value (SUV) and metabolic tumor volume (MTV) have been reported to have important prognostic and predictive roles [11–15]. However, the SUV is easily affected by multiple factors (e.g., blood glucose level, body weight, scanning protocol, reconstruction parameters, and dose extravasation [16,17]), and it can only provide information on tumor glycolysis. The MTV is a parameter that can reflect both tumor radioactive uptake and tumor volume simultaneously, and the total MTV is a semiquantitative interpretation of the whole-body tumor burden. However, to date, the approach of MTV measurement has not been standardized, and the value of the MTV varies based on different measurement procedures [12].

Intratumor heterogeneity, which correlates with tumor aggressiveness and poor prognosis, has been increasingly noted [18]. As a noninvasive way to assess intratumor heterogeneity, texture analysis (TA) is able to extract a series of quantitative features from routine medical images through a variety of mathematical methods [19]. PET TA has been reported to have potential in various tumors [20]. In lymphomas, the roles of PET TA in differential diagnosis [21], treatment response prediction, and prognosis have been studied [22–29]. However, based on the limited evidence and the indeterminate choices of PET texture features, the value of PET TA in lymphoma remains unclear. Moreover, to the best of our knowledge, the application of PET TA in PGIL has been reported by only one study, which did not include intestinal PGIL-DLBCL [30].

In the present study, we aimed to explore whether PET/CT TA was useful in predicting interim response in PGIL-DLBCL patients treated with chemotherapy and to compare the predictive values of texture features with those of the maximum standard uptake value (SUVmax) and MTV. We also aimed to obtain a prediction probability using texture features, clinical characteristics, and traditional PET semiquantitative features.

## 2. Materials and Methods

### 2.1. Patients

**Figure 1:**
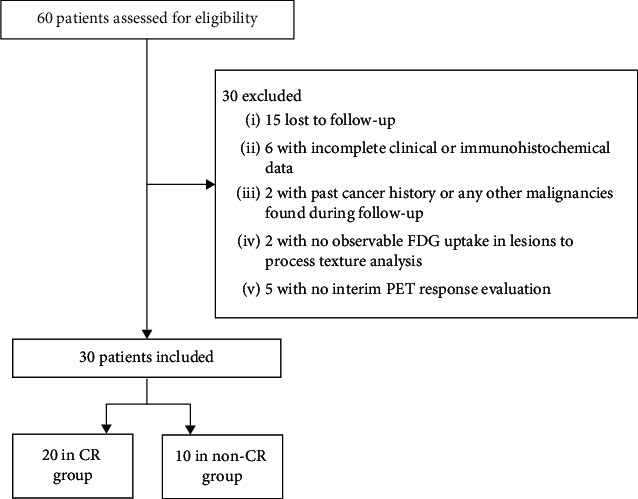
Flowchart of patient inclusion and exclusion. The flowchart shows information about the inclusion and exclusion criteria that were used to ultimately include 30 patients with PGIL-DLBCL, including 20 in the CR group and 10 in the non-CR group.

This retrospective study was approved by the local ethics committee, and the requirement for informed consent was waived. From June 2013 to March 2019, 60 patients with newly diagnosed PGIL-DLBCL were retrospectively reviewed. The inclusion criteria were as follows: (1) a diagnosis of PGIL-DLBCL confirmed by biopsy, (2) ^18^F-FDG PET/CT scan before treatment, and (3) an interval between the PET/CT scan and biopsy of less than 1 month. The exclusion criteria were as follows: (1) loss of follow-up (*n* = 15), (2) incomplete clinical or immunohistochemical data (*n* = 6), (3) past cancer history or any other malignancies found during follow-up (*n* = 2), (4) no observable FDG uptake in lesions to process TA (*n* = 2), and (5) no interim PET response evaluation (*n* = 5). The flowchart of patient inclusion and exclusion is shown in Figure 1.

### 2.2. Clinicopathological Data and Therapeutic Response Evaluation

Clinical and pathological information, including the involved sites in the GI tract, Lugano stage, international prognostic index (IPI), histological subtypes (germinal center B-cell-like (GCB) and non-GCB), and Ki67, was collected retrospectively from inpatient medical records and histologic reports.

The treatment plan for all eligible patients was 6–8 cycles of chemotherapy. A PET/CT scan was performed after 3-4 cycles of chemotherapy (29 after 4 cycles, 1 after 3 cycles) to evaluate the interim response. The interim response was assessed according to the PET-CT-based Lugano response criteria [9]. A complete response was defined as a score of 1–3 with or without a residual mass based on the Deauville 5-point scale and no evidence of FDG-avid disease in the marrow.

### 2.3. ^18^F-FDG PET/CT Image Acquisition

^18^F-FDG PET/CT scans were performed with a 16-row hybrid PET/CT scanner (Gemini GXL16, Philips Medical System, Cleveland, Ohio, USA). The serum glucose levels of all patients were confirmed to be less than 11.1 mmol/L after fasting for at least 6 hours. Then, 5.2 MBq (±10%) per kilogram of body weight of ^18^F-FDG was injected intravenously 50–90 minutes before PET/CT scanning. All patients were encouraged to drink 600–1000 ml of water 5 minutes before scanning to achieve gastric distension and were scanned in the supine position with arms elevated above the head and breathing at rest. For each patient, an unenhanced CT from the skull base to the upper thigh was performed for anatomic information and attenuation correction (CT scanning parameters: 50 mA, 120 kV, 5 mm section thickness, 5 mm increment, and a pitch of 0.813). The CT images were reconstructed to a 512 × 512 matrix. A 3-dimensional PET scan of the same region was subsequently obtained without any change in position. The emission data were acquired for 70 seconds per bed position, and a total of 8-9 bed positions were performed. The PET images were reconstructed in a 144 × 144 matrix with a voxel size of 4 mm × 4 mm × 4 mm and a slice thickness of 4 mm by a line-of-response algorithm using Syntegra software (Philips Corp., Amsterdam, Netherlands).

### 2.4. Image Interpretation

All PET/CT images were retrospectively reviewed by a radiologist (Y. S., with 9 years of experience in oncologic PET/CT) and confirmed by another radiologist (C. J., with 6 years of experience in oncologic PET/CT). Both radiologists had no knowledge of the results of the interim response assessment. Since we did not aim to explore the diagnostic value of PET/CT in DLBCL-PGIL, the tumor location was not blinded. The PET/CT images were transferred to the MedEx workstation (Beijing, China) to measure the SUVmax and MTV. The SUVmax and MTV were automatically generated by the MedEx workstation after each tumor was enclosed in a cropping sphere, and the MTV was defined as the volume of voxels with SUVs higher than the threshold of 41% × SUVmax.

### 2.5. Texture Analysis

The PET and CT images were uploaded to in-house software (Image Analyzer 2.0, China), and TA was performed separately on PET and CT images. In cases with multiple tumors in the GI tract, the tumor with the highest SUVmax was chosen for analysis.

In the PET images, regions of interest (ROIs) were manually drawn slice by slice to cover the entire volume of the tumors. The GI lumen and adjacent lesions (such as involved lymph node or liver tissue) were carefully avoided. The following first- and second-order texture features were derived from the PET images, including (1) first-order features: mean, standard deviation (SD), max-frequency, mode, minimum, maximum, cumulative percentiles (the 5th, 10th, 25th, 50th, 75th, and 90th percentiles), skewness, kurtosis, entropy, volume, and max-diameter and (2) local textural features of the grey-level co-occurrence matrix (GLCM): entropy_GLCM_, energy_GLCM_, inertia_GLCM_, and variance_GLCM_.

In each CT image, an ROI was manually drawn along the margin of the tumor on the section that depicted the largest area of the lesion, with artefacts and the gastrointestinal lumen carefully avoided. The attenuation value of each pixel within the ROIs was automatically read and analyzed by the software, and the following texture features were generated from CT images: mean, SD, max-frequency, mode, maximum, minimum, skewness, kurtosis, entropy, max-diameter, entropy_GLCM_, energy_GLCM_, inertia_GLCM_, and variance_GLCM_.

### 2.6. Statistical Analysis

The Shapiro–Wilk normality test was applied to evaluate the distribution characteristics of the SUVmax, MTV, PET texture parameters, and CT texture parameters. The differences in the CR rate in patients with different clinicopathological characteristics were compared by Fisher's exact test. Feature selection was processed by two steps: (1) univariate filtering was performed on all of the texture features using the Mann–Whitney *U* test, and features without significant differences between the CR and non-CR groups were eliminated; (2) the remaining features were classified into the following four categories: (a) features describing FDG uptake intensity; (b) features describing the distribution of grey-level intensity; (c) features describing tumor size; and (d) features describing intratumor heterogeneity. In each category, the feature with the lowest *p* value in the previous step was selected. For the SUVmax, MTV, and each feature selected as previously described, a receiver operating characteristic (ROC) analysis was performed to evaluate the efficacy in distinguishing between the CR and non-CR group. A binary logistic regression analysis was performed to generate a prediction probability. The ROC analysis was used to assess the distinguishing efficacy of the prediction probability. The consistency between the MTV and volume was assessed by the Wilcoxon signed rank test. The interobserver agreement in the measurement of PET and CT texture parameters was estimated with the intraclass correlation coefficient (ICC; 0.000–0.200, poor; 0.201–0.400, fair; 0.401–0.600, moderate; 0.601–0.800, good; and 0.801–1.000, excellent). The ROC analysis was performed with Med-Calc Statistical Software version 19.0.7 (Med-Calc Software bvba, Ostend, Belgium; http://www.medcalc.org; 2015), and other statistical analyses were performed with SPSS (version 22.0 for Microsoft Windows ×64, SPSS, Chicago, IL, USA). A two-tailed *p* value <0.05 was considered statistically significant.

## 3. Results

### 3.1. Patients and Clinicopathological Characteristics

A total of 30 patients were ultimately included in our study cohort (11 males, 19 females; age range, 31–79 years; median age, 56 years; interquartile range, 47–63 years).

Among the 30 enrolled patients, 25 were treated with the R–CHOP protocol, while 5 were treated with other protocols that included rituximab. Two patients underwent PET/CT response assessments after 4 cycles of chemotherapy and then dropped out of the treatment plan (one died from severe interstitial pneumonia, and one turned to traditional Chinese medical therapy). Since the withdrawals were not expected at the time when they accepted the PET/CT response assessments, they were still considered to be “interim responses.”

In the PET/CT interim response assessment, 20 patients achieved CR (three with Deauville score 1, eight with Deauville score 2, and nine with Deauville score 3), while 10 patients did not achieve CR (three with Deauville score 4 and seven with Deauville score 5). The patients' clinicopathological characteristics are presented in Table 1.

### 3.2. Clinicopathological Characteristics for Interim Response Prediction

The CR rates of different groups of stages, IPI scores, histological subtypes, involved sites, and Ki67 are shown in Table 2. To find an optimal cutoff value for Ki67, the investigators tried 60%, 70%, 80%, and 90% successively, finding the *p* value to be the lowest when using 80% as a cutoff. Thus, the cohort was divided into groups with Ki67 < 80% and Ki67 ≥ 80%. The CR rate was significantly lower in patients with intestinal involvement. Although the CR rates were lower in patients with higher Lugano stage, IPI score, and Ki67 score, the differences were not statistically significant.

### 3.3. Feature Selection

Some texture features did not have a normal distribution. The detailed results of the normality test are shown in Supplemental Table 1.

In the first step of feature selection, a total of 17 PET texture features and 24 CT texture features were found to be of no significant differences between the CR and non-CR groups and were eliminated. The detailed results of the Mann–Whitney *U* test of these features are shown in Supplemental Table 2.

Among the remaining PET texture features, the mean, SD, max-frequency, 50^th^ percentile, 75^th^ percentile, 90^th^ percentile, maximum, entropy, volume, max-diameter, entropy_GLCM10_, and entropy_GLCM12_ were significantly lower in the CR group, while the energy_GLCM10_, energy_GLCM11_, energy_GLCM12_, and energy_GLCM13_ were significantly higher in the CR group. The remaining CT texture features included the max-frequency and max-diameter, which were significantly lower in the CR group. The SUVmax and MTV were also significantly lower in the CR group (Table 3).

In the second step of feature selection, the remaining features were categorized and selected as follows. (a) Among the features describing FDG uptake intensity, including the mean, 50^th^ percentile, 75^th^ percentile, 90^th^ percentile, and maximum (*p* values were 0.028, 0.031, 0.028, 0.035, and 0.039, respectively), the mean and the 50^th^ percentile had the lowest *p* values. Because the grey-level intensities were not normally distributed, the 50^th^ percentile was selected in this category. (b) Among the features describing the distribution of grey-level intensity, including the SD, max-frequency, and CT max-frequency (*p* values were 0.044, 0.019 and 0.011, respectively), the CT max-frequency was selected. (c) Among the features describing tumor size, including the volume, max-diameter, and CT max-diameter (*p* values were 0.006, 0.011, and 0.024, respectively), the volume was selected. (d) Among the features describing intratumor heterogeneity, including first-order entropy, entropy_GLCM10_, entropy_GLCM12_, energy_GLCM10_, energy_GLCM11_, energy_GLCM12_, and energy_GLCM13_ (*p* values were 0.007, 0.015, 0.011, 0.049, 0.039, 0.035, and 0.039, respectively), the first-order entropy was selected.

### 3.4. SUVmax, MTV, and Texture Features for Interim Response Prediction

**Figure 2:**
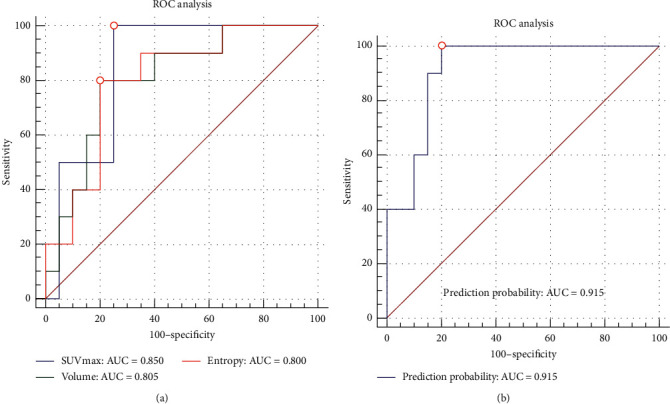
ROC analysis of parameters with AUCs ≥0.800 and the prediction probability. (a) ROC analysis of the SUVmax, volume, and entropy. The AUCs were 0.850, 0.805, and 0.800, respectively. (b) ROC analysis of the prediction probability generated from the combination of the SUVmax, entropy, volume, and intestinal involvement. The AUC was 0.915.

The predictive values of the SUVmax, MTV, and selected texture features for interim response were evaluated by ROC analyses, and the results are displayed in Table 4. The areas under the curves (AUCs) of the SUVmax and MTV for distinguishing the non-CR group from the CR group were 0.850 and 0.790, respectively. Among the texture features selected, the AUCs of the first-order entropy and volume of the PET images were ≥0.80 (0.800 and 0.805, respectively). The ROCs of the parameters with AUCs ≥0.800 are displayed in Figure 2(a).

### 3.5. Binary Logistic Regression Analysis

Intestinal involvement and the SUVmax, volume, and entropy were selected to be included to generate a prediction probability. The Hosmer–Lemeshow test showed a chi-square value of 9.727 and a *p* value of 0.285. The prediction probability showed an AUC of 0.915 in the ROC analysis (sensitivity = 1.00, specificity = 0.80, accuracy = 0.87) (Figure 2(b)).

### 3.6. Consistency between the MTV and Volume

The median MTV and median volume were 45.20 cm^3^ (interquartile range = 13.88–127.45 cm^3^) and 59.87 cm^3^ (interquartile range = 20.66–260.91 cm^3^), respectively. There was a significant difference between the MTV and volume according to the Wilcoxon signed rank test (*Z* = −3.834, *p* < 0.001). The AUCs of MTV and volume were not significantly different (*Z* = 0.227, *p* = 0.821).

### 3.7. Interobserver Agreement

The SUVmax, MTV, some PET texture features (mean, SD, maximum, 90^th^ percentile, 75^th^ percentile, 50^th^ percentile, max-frequency, volume, max-diameter, entropy, inertia_GLCM11_, inertia_GLCM12_, inertia_GLCM13_, variance_GLCM10_, variance_GLCM11_, variance_GLCM12_, and variance_GLCM13_), and some CT features (max-frequency, max-diameter, entropy_GLCM13_, inertia_GLCM10_, inertia_GLCM11_, inertia_GLCM12_, and inertia_GLCM13_) showed excellent interobserver agreement. Other PET and CT texture features showed good-to-poor interobserver agreement. The detailed ICCs are shown in Supplemental Table 3.

## 4. Discussion

The present study explored the use of the SUVmax, the MTV, PET/CT texture features, and clinicopathological characteristics in predicting the interim treatment response of PGIL-DLBCL. We found that the SUVmax, the MTV, some texture features, and the tumor location were useful parameters in interim response prediction. Moreover, employing a combination of the pretreatment SUVmax, texture features (entropy and volume), and intestinal involvement further improved the predictive value in PGIL-DLBCL patients.

Previous studies have demonstrated that a high SUV is associated with a poor prognosis [15,31]. As a routinely used semiquantitative parameter in ^18^F-FDG PET/CT that reflects tumor glucose metabolism, the SUV is associated with tumor aggressiveness. We consistently found that the SUVmax was significantly higher in the non-CR group and that the SUVmax had the highest AUC (0.850) in predicting the interim treatment response among all single parameters. These results further confirmed the SUVmax as an excellent predictor of the interim response of PGIL-DLBCL.

PET TA provides information about the intratumor heterogeneity of FDG uptake noninvasively from routine images [32]. The first-order texture features based on histogram and second-order texture features calculated using the GLCM are the most widely used texture features in oncological PET/CT images, with most of them reported to be robust [19, 33]. The first-order texture features describe the global grey-level intensity distribution inside a tumor, which provides an overall view of the data. The GLCM features describe the spatial relationships of pairs of pixels or voxels with certain grey-level intensities, in certain directions and with certain distances between them [34]. Among the commonly used GLCM features, entropy_GLCM_ describes the extent of disorder, energy_GLCM_ describes the uniformity of grey-level voxel pairs, inertia_GLCM_ (also called contrast in some studies) describes the local variation, and variance_GLCM_ describes the degree of dispersion [33, 35]. Additionally, the first-order features and GLCM features were reported to be more reproducible than some other texture features derived from other matrices (e.g., the grey-level intensity size zone matrix) [36]. Thus, the investigators selected first-order features and GLCM features (entropy_GLCM_, energy_GLCM_, inertia_GLCM_, and variance_GLCM_) to be analyzed.

In the current study, some first-order texture features, including the mean, 50^th^ percentile, 75^th^ percentile, 90^th^ percentile, and maximum, were found to be significantly higher in the non-CR group, with AUCs ranging from 0.735 to 0.750. These features reflect the degrees of FDG uptake of the pixels and provide detailed information on FDG distribution.

The energy of GLCM is calculated by the formula ∑_*i*_∑_*j*_*P*_*d*_^2^(*i*, *j*), which measures the number of repeated pairs of pixels [37]. The more often the pairs of pixels (*i*, *j*) with certain intensities and spatial relationships co-occur, the higher the energy is. A previous study including PET/CT images of 35 primary gastric DLBCL patients reported that energy_GLCM_ failed to predict either the progression-free survival (PFS) or the overall survival (OS) [30]. However, this study focused on the prediction of the prognosis rather than the treatment response. In the current study, which focused on the predictive value of PET/CT texture features for the interim response of PGIL-DLBCL, energy_GLCM10_-_13_ was found to be significantly lower in the non-CR group, with AUCs ranging from 0.725 to 0.738. These results suggested that the PET images of the non-CR group were less patterned.

Entropy quantitatively characterizes the intratumor heterogeneity. The more chaotically the intensities of the pixels are distributed, the higher the entropy [37]. A previous study of PET images of 82 patients with aggressive NHL found that entropy was unable to predict the treatment response or prognosis [22]. However, this study was performed in a heterogeneous cohort, and the treatment response was evaluated at the end of chemotherapy. Another study of CT images of 100 patients with HL reported that the mean value and entropy of entropy_GLCM_ decreased significantly after 2–4 cycles of chemotherapy compared to the baseline, indicating decreased tissue heterogeneity during the treatment [38]. The current study found that the non-CR group in the interim response evaluation had significantly higher first-order entropy, entropy_GLCM10_, and entropy_GLCM12_ (the *p* values were 0.007, 0.015, and 0.011, respectively; the AUCs were 0.800, 0.775, and 0.785, respectively). Entropy and energy, which describe intratumor heterogeneity from different perspectives, could be complements to each other. Among all of the texture features being analyzed, the first-order entropy had the highest AUC. Although the AUCs of the texture features were not as high as that of the SUVmax, there were no significant differences between them. Thus, the texture features mentioned above were good complementary predictors of the interim response.

In the present study, a high volume and high MTV were found to be predictors of non-CR. The volume and MTV are similar parameters that indicate the tumor burden but are measured by different methods (the volume was derived from the manually drawn ROI, while the MTV was generated automatically by a computer program based on a set threshold). Multiple studies have demonstrated that a high MTV is associated with an insufficient treatment response and a poor prognosis in lymphoma [11–13, 24, 39]. Accordingly, we found that non-CR patients had significantly higher volumes and MTVs than CR patients. Due to the difference in measurement methods, the volume was found to be larger than the MTV. Generally, the volume has been thought to be less reproducible than the MTV, while the volume has advantages in contouring irregular tumors and avoiding the incorrect exclusion of low FDG-avid regions. In the present study, the MTV had a higher ICC than the volume, while both of them showed excellent interobserver agreement. The AUC in the ROC analysis of the volume was slightly higher than that of the MTV, but there was no significant difference between them. According to these results, despite the difference in the values of the volume and MTV, they were both useful features in predicting the interim response of PGIL-DLBCL.

In addition, intestinal involvement was found to be a predictor of non-CR in the present study. Previous studies have reported poorer prognoses of intestinal lymphoma than gastric lymphoma [40, 41]. This finding was attributed to intestinal lymphoma presenting more aggressive subtypes [41]. Although the current study concerned only one single aggressive subtype (DLBCL), patients with intestinal involvement were still found to have a lower CR rate. Additionally, Ishikawa et al. [40] reported that PD-L1 expression on microenvironment immune cells impacted the prognosis of PGIL-DLBCL. Thus, the difference in the treatment response between patients with and without intestinal involvement might be associated with factors beyond tumor histology, and the microenvironment might be an important factor.

The SUVmax, entropy, volume, and intestinal involvement were chosen and combined to generate a prediction probability. This combination characterized the tumors from different perspectives, namely, glucose metabolism, intratumor heterogeneity, tumor burden, and anatomical site. The prediction probability was demonstrated to be an excellent predictor of the interim response with an AUC higher than any single parameter (AUC = 0.915).

The interobserver ICCs were calculated to evaluate the reproducibility of the texture features. The SUVmax, MTV, mean, SD, maximum, higher percentiles (50^th^, 75^th^, and 90^th^), first-order entropy, volume, and max-diameter of PET images were found to be of excellent interobserver reproducibility, with ICCs ranging from 0.807 to 0.988. However, the first-order skewness and kurtosis had relatively low ICCs (0.515 and 0.430, respectively). These results accorded with those of a previous study [36]. In contrast to the previous study, the reproducibility of entropy_GLCM_ and energy_GLCM_ was poor to moderate; this might be caused by the technical differences between the different computer programs used for TA. Additionally, the interobserver reproducibility was tested by 5 observers in the previous study and by only 2 observers in the present study, which might have an impact on the results of the ICCs. Despite the diversity in the interobserver reproducibility of different texture features, the features that were found to be of excellent predictive value for the interim response and were chosen to generate the prediction probability were all found to have excellent interobserver agreements in the current study (SUVmax, first-order entropy, and volume; the ICCs were 0.936, 0.864, and 0.898, respectively).

The current study has several limitations. First, the present study was preliminary and retrospective. The study cohort was small, as it was limited by the low incidence of PGIL, the filtered histological subtype of DLBCL, and the exclusion of patients who did not accept consecutive chemotherapy and PET/CT scans. Some PET/CT scans were performed beyond the recommended interval between FDG administration and acquisition [42] for unexpected reasons (such as machine malfunction, mobility-impaired patients, and the necessary extension of the scan field or delayed scan of previous patients), which could have affected the measurement of the SUVmax and MTV. Interim response assessments were performed after 3-4 cycles of chemotherapy according to the previous clinical protocol of DLBCL patient management, while there was increasing evidence of the benefit of early (after 2 cycles of chemotherapy) PET-adapted therapy [43, 44]. Thus, a prospective study of early response assessment with an enlarged sample size and PET/CT scans executed strictly according to the standard protocol should be performed in the future. Second, due to the limited size of the study cohort, it was difficult to separate some patients into a validation subset. Thus, it is very important to perform validation analysis with external data in the future. Third, there was a discrepancy in sample sizes between the CR and non-CR groups (20 vs. 10), which was mainly caused by the inherent treatment outcome of the current chemotherapy strategies. The statistical results could be affected. An increased sample size and specialized statistical techniques should be used in future studies.

## 5. Conclusions

The preliminary study indicated that TA had potential for improving the value of pretreatment PET/CT in predicting the interim response in PGIL-DLBCL. However, prospective studies with increased sample sizes and validation analyses should be performed to confirm the present findings.

## Figures and Tables

**Table 1:** Clinicopathological characteristics.

Characteristics	Number of patients (*n* = 30)
Gender	
Male	11
Female	19
Age (years)	
≤60	21
>60	9
Lugano stage	
Stage I	10
Stage II	9
Stage IV	11
Number of lesion(s) in GI tract	
One	26
Two or more than two	4
Involved sites in GI tract	
Fundus of stomach	1
Body of stomach	14
Antrum of stomach	11
Duodenum	4
Jejunum or ileum	3
Ileocecal junction	4
Colon	4
Histological subtype	
GCB-DLBCL	13
Non-GCB-DLBCL	17
IPI score	
0	9
1	9
2	3
3	7
4	2
5	0
Interim response evaluation	
CR	20
Non-CR	10

GI: gastrointestinal; GCB-DLBCL: germinal center B-cell-like diffused large B-cell lymphoma; IPI: international prognostic index; CR: complete remission.

**Table 2:** CR rates in patients with different clinicopathological characteristics.

Characteristic	Number of CR	Number of non-CR	CR rate (%)	*p* value
Lugano stage				
Stage I	9	1	90.0	0.062
Stage II and IV	11	9	50.5	

IPI score				
0–2	16	5	76.2	0.104
3–5	4	5	44.4	

Histological subtype				
GCB	9	4	69.2	0.554
Non-GCB	11	6	64.7	

Intestinal involvement				
Involved	6	7	46.2	0.045^*∗*^
Not involved	14	3	82.4	

Ki67				
<80%	9	3	75.0	0.350
≥80%	11	7	61.1	

^*∗*^ *p* < 0.05 indicates a statistically significant difference between groups. GCB: germinal center B-cell-like.

**Table 3:** Differences between the CR group and non-CR group.

Parameter	Median (interquartile range)		*p* value
	CR group	Non-CR group	
SUVmax	15.15 (8.73–21.75)	23.95 (21.45–29.03)	0.001

MTV(cm^3^)	17.80 (11.70–53.08)	145.10 (66.63–613.90)	0.009

PET texture features			
Mean	3983.04 (3024.75–7348.02)	7348.96 (5402.80–10440.45)	0.028
SD	1781.05 (1042.33–2554.85)	2878.15 (1933.98–5193.57)	0.044
Max-frequency	3.00 (2.00–4.00)	5.00 (3.00–7.25)	0.019
Maximum	9324.50 (6768.75–16080.00)	17682.00 (11709.25–24430.00)	0.031
50^th^ percentile	3705.50 (2620.50–6841.25)	6699.00 (4841.00–9395.25)	0.028^*∗*^
75^th^ percentile	4789.00 (3903.75–9199.00)	8963.50 (6721.50–13698.00)	0.035
90^th^ percentile	6081.00 (5408.50–10572.75)	10257.00 (8360.00–16868.50)	0.039
Entropy	6.3777 (5.4630–7.0874)	7.7240 (7.0291–8.5527)	0.007^*∗*^
Volume (mm^3^)	41504 (15920–85760)	226272 (83760–683088)	0.006^*∗*^
Max-diameter (mm)	48.44 (36.50–75.96)	85.62 (68.95–142.09)	0.011
Entropy_GLCM10_	9.02 (7.16–9.76)	11.45 (8.51–12.56)	0.015
Entropy_GLCM12_	9.07 (7.36–9.84)	11.38 (8.70–12.56)	0.011
Energy_GLCM10_	0.001880 (0.000961–0.005434)	0.000363 (0.000172–0.002750)	0.049
Energy_GLCM11_	0.001550 (0.000863–0.004614)	0.000364 (0.000167–0.002392)	0.039
Energy_GLCM12_	0.001757 (0.000951–0.005436)	0.000388 (0.000172–0.002414)	0.035
Energy_GLCM13_	0.001544 (0.000821–0.004535)	0.000338 (0.000167–0.002186)	0.039

CT texture features			
Max-frequency	31.50 (23.75–81.00)	99.50 (46.00–276.25)	0.011^*∗*^
Max-diameter (mm)	52.05 (40.58–89.65)	75.15 (64.88–124.18)	0.024

^*∗*^The features selected in the second step of feature selection.

**Table 4:** ROC analysis of SUVmax, MTV, and texture features.

Parameter	Cutoff	Sensitivity	Specificity	Accuracy	AUC	*p* value
SUVmax	18.6	1.00	0.75	0.83	0.850	<0.001

MTV (cm^3^)	49.7	0.90	0.75	0.80	0.790	0.006

PET texture features						
50^th^ percentile	4139	0.90	0.70	0.77	0.750	0.012
Entropy	7.13	0.80	0.80	0.80	0.800	<0.001
Volume (mm^3^)	85824	0.80	0.80	0.80	0.805	<0.001

CT texture features						
Max-frequency	44	0.90	0.70	0.77	0.783	0.001

## Data Availability

The data used to support the findings of this study are available from the corresponding author upon request.

## Supplementary Materials

Supplementary Materials Table 1: normality test of SUV and texture features. Table 2: features without significant differences between the CR and non-CR groups in the Mann–Whitney *U* test. Table 3: interobserver agreement of PET texture features.
