# Supplementary material for: Texture Analysis Improves the Value of Pretreatment 18F-FDG PET/CT in Predicting Interim Response of Primary Gastrointestinal Diffuse Large B-Cell Lymphoma
Source: Contrast Media Mol Imaging. 2020 Aug 21;2020:2981585. doi: 10.1155/2020/2981585 (PMC7463417; doi:10.1155/2020/2981585)
Supplement: Supplementary Materials — Table 1: normality test of SUV and texture features. Table 2: features without significant differences between the CR and non-CR groups in the Mann–Whitney U test. Table 3: interobserver agreement of PET texture features. [file 2981585.f1.docx]

Table 1: Normality test of SUV and texture features

| Parameter | Mean | SD | Median | p value |
| --- | --- | --- | --- | --- |
| SUVmax | 18.41 | 8.17 | 18.85 | 0.545 |
| MTV | 153.61 | 262.97 | 45.20 | ＜0.001* |
| PET texture features |  |  |  |  |
| mean | 5900.71 | 3354.10 | 4531.40 | 0.024* |
| SD | 2561.74 | 1860.99 | 1966.46 | 0.001* |
| max-frequency | 4.03 | 2.43 | 3.00 | ＜0.001* |
| mode | 3608.50 | 2481.90 | 2833.50 | 0.001* |
| minimum | 1519.33 | 663.95 | 1453.50 | 0.298 |
| maximum | 13653.63 | 8591.43 | 11796.50 | 0.015* |
| 5^th^ percentile | 2593.83 | 1288.71 | 2568.50 | 0.056 |
| 10^th^ percentile | 2934.37 | 1473.04 | 2800.50 | 0.080 |
| 25^th^ percentile | 3814.13 | 2067.75 | 3407.50 | 0.045* |
| 50^th^ percentile | 5465.77 | 3185.22 | 4233.50 | 0.018* |
| 75^th^ percentile | 7614.20 | 4623.91 | 5951.50 | 0.008* |
| 90^th^ percentile | 9591.10 | 5941.61 | 7863.50 | 0.006* |
| skewness | 0.5749 | 0.5431 | 0.5245 | 0.383 |
| kurtosis | 2.92 | 1.23 | 2.66 | ＜0.001* |
| entropy | 6.81 | 1.41 | 6.68 | 0.599 |
| volume | 228425.60 | 376085.59 | 59872.00 | ＜0.001* |
| area | 57115.73 | 94067.43 | 14968.00 | ＜0.001* |
| max-diameter | 75.01 | 46.27 | 58.45 | 0.001* |
| entropy_GLCM10_ | 9.20 | 2.61 | 9.10 | 0.033* |
| entropy_GLCM11_ | 8.65 | 3.01 | 9.22 | 0.001* |
| entropy_GLCM12_ | 9.22 | 2.64 | 9.22 | 0.019* |
| entropy_GLCM13_ | 8.68 | 2.97 | 9.26 | 0.001* |
| energy_GLCM10_ | 0.004335 | 0.01132 | 0.001590 | ＜0.001* |
| energy_GLCM11_ | 0.002917 | 0.006086 | 0.001371 | ＜0.001* |
| energy_GLCM12_ | 0.004513 | 0.01288 | 0.001551 | ＜0.001* |
| energy_GLCM13_ | 0.003375 | 0.008262 | 0.001271 | ＜0.001* |
| inertia_GLCM10_ | 33150.08 | 20018.08 | 27553.75 | 0.059 |
| inertia_GLCM11_ | 21122.35 | 11640.74 | 19073.35 | 0.058 |
| inertia_GLCM12_ | 30150.98 | 14609.29 | 27925.60 | 0.003* |
| inertia_GLCM13_ | 18609.47 | 12505.46 | 16435.75 | ＜0.001* |
| variance_GLCM10_ | 48205.10 | 18543.39 | 50529.10 | 0.957 |
| variance_GLCM11_ | 48077.92 | 18543.39 | 50529.05 | 0.981 |
| variance_GLCM12_ | 47797.43 | 17959.49 | 50651.75 | 0.977 |
| variance_GLCM13_ | 48613.13 | 18557.74 | 49963.00 | 0.982 |
| CT texture features |  |  |  |  |
| mean | 43.31 | 4.27 | 43.48 | 0.410 |
| SD | 11.03 | 1.90 | 11.16 | 0.245 |
| max-frequency | 92.43 | 99.77 | 45.00 | ＜0.001* |
| mode | 44.27 | 5.21 | 45.00 | 0.206 |
| minimum | -5.27 | 19.34 | -6.00 | 0.031* |
| maximum | 75.03 | 7.43 | 74.00 | 0.513 |
| skewness | -0.48 | 0.38 | -0.36 | 0.070 |
| kurtosis | 4.05 | 1.40 | 3.69 | ＜0.001* |
| entropy | 3.73 | .0162 | 3.78 | 0.037* |
| max-diameter | 73.52 | 39.03 | 65.35 | 0.009* |
| entropy_GLCM10_ | 6.53 | 0.44 | 6.56 | 0.164 |
| entropy_GLCM11_ | 6.51 | 0.43 | 6.54 | 0.214 |
| entropy_GLCM12_ | 6.56 | 0.43 | 6.61 | 0.132 |
| entropy_GLCM13_ | 6.32 | .044 | 6.33 | 0.200 |
| energy_GLCM10_ | 0.01551 | 0.00508 | 0.01468 | 0.034* |
| energy_GLCM11_ | 0.01552 | 0.00479 | 0.01478 | 0.088 |
| energy_GLCM12_ | 0.01510 | 0.00478 | 0.01425 | 0.054 |
| energy_GLCM13_ | 0.01792 | 0.00583 | 0.01682 | 0.033* |
| inertia_GLCM10_ | 9.40 | 3.51 | 8.64 | 0.055 |
| inertia_GLCM11_ | 8.48 | 3.20 | 7.90 | 0.065 |
| inertia_GLCM12_ | 10.16 | 3.65 | 10.06 | 0.022* |
| inertia_GLCM13_ | 5.38 | 1.98 | 5.13 | 0.216 |
| variance_GLCM10_ | 7.07 | 2.06 | 7.33 | 0.028* |
| variance_GLCM11_ | 7.11 | 2.06 | 7.27 | 0.033* |
| variance_GLCM12_ | 7.08 | 2.06 | 7.26 | 0.036* |
| variance_GLCM13_ | 7.14 | 2.10 | 7.30 | 0.018* |

*p value＜0.05 means that the parameter does not comply with the normal distribution.

Table 2: Features without significant differences between the CR and non-CR groups in Mann-Whitney U test

| Parameter | Median (Interquartile range) | | P value |
| --- | --- | --- | --- |
|  | CR group | Non-CR group |  |
| PET texture features |  |  |  |
| mode | 2554.00 (1612.00-4099.50) | 3530.50 (2732.25-6839.00) | 0.120 |
| minimum | 1380.50 (1037.25-1925.75) | 1571.00 (943.00-2254.00) | 0.888 |
| 5^th^ percentile | 2208.00 (1509.75-3024.00) | 2847.50 (2444.50-3860.50) | 0.109 |
| 10^th^ percentile | 2465.00 (1633.00-3397.75) | 3198.00 (2857.00-4696.25) | 0.074 |
| 25^th^ percentile | 2929.00 (1921.50-4552.00) | 4337.50 (3529.00-7111.75) | 0.061 |
| skewness | 0.6122 (0.1725-0.9351) | 0.3433 (0.1530-0.8999) | 0.475 |
| kurtosis | 2.7189 (2.2190-3.1472) | 2.5127 (2.0900-3.0814) | 0.588 |
| entropy_GLCM11_ | 9.22 (7.62-9.98) | 9.66 (7.00-11.76) | 0.448 |
| entropy_GLCM13_ | 9.26 (7.62-10.06) | 9.71 (7.09-11.73) | 0.448 |
| inertia_GLCM10_ | 25208.40 (20710.65-49770.58) | 32441.05 (18537.23-43549.58) | 0.88 |
| inertia_GLCM11_ | 17567.90 (10492.43-28488.80) | 23874.25 (11384.53-29417.65) | 0.713 |
| inertia_GLCM12_ | 27711.30 (18859.80-38770.98) | 27925.60 (21047.90-34294.78) | 0.779 |
| inertia_GLCM13_ | 16696.40 (9548.72-19875.00) | 16435.75 (14816.43-22286.95) | 0.619 |
| variance_GLCM10_ | 49053.40 (36818.75-56416.28) | 53956.70 (31412.10-76656.43) | 0.53 |
| variance_GLCM11_ | 49715.75 (37124.78-55015.28) | 53418.80 (30814.80-74789.20) | 0.53 |
| variance_GLCM12_ | 50651.75 (34434.40-55256.73) | 53070.70 (31906.50-73069.38) | 0.559 |
| variance_GLCM13_ | 49119.70 (35778.18-55942.18) | 55931.30 (32821.80-78107.55) | 0.397 |
| CT texture features |  |  |  |
| mean(HU) | 42.70 (40.86-44.59) | 47.30 (40.97-48.78) | 0.120 |
| SD | 11.87 (9.64-12.93) | 10.49 (8.65-11.51) | 0.143 |
| mode (HU) | 43.00 (40.25-47.00) | 47.00 (39.50-52.00) | 0.225 |
| minimum (HU) | -3.50 (-17.25-12.25) | -6.00 (-16.75-8.00) | 0.754 |
| maximum (HU) | 74.00 (69.25-78.75) | 74.00 (69.00-81.50) | 0.888 |
| skewness | -0.4286 (-0.8188- -0.1488) | -0.3562 (-0.8087- -0.2056) | 0.983 |
| kurtosis | 3.72 (3.12-4.22) | 3.51 (3.23-4.93) | 0.812 |
| entropy | 3.81 (3.62-3.88) | 3.69 (3.54-3.83) | 0.307 |
| entropy_GLCM10_ | 6.56 (6.30- 6.96) | 6.49 (5.97-6.96) | 0.650 |
| entropy_GLCM11_ | 6.54 (6.30- 6.96) | 6.43 (5.99- 6.96) | 0.779 |
| entropy_GLCM12_ | 6.61 (6.30- 6.93) | 6.48 (6.04- 6.99) | 0.746 |
| entropy_GLCM13_ | 6.33 (6.15- 6.73) | 6.22 (5.70- 6.71) | 0.475 |
| energy_GLCM10_ | 0.01468 (0.01109-0.01745) | 0.01501 (0.01118-0.02178) | 0.681 |
| energy_GLCM11_ | 0.01478 (0.01106-0.01719) | 0.01582 (0.01129-0.02102) | 0.681 |
| energy_GLCM12_ | 0.01425 (0.01091-0.01702) | 0.01551 (0.01117-0.02031) | 0.681 |
| energy_GLCM13_ | 0.01682 (0.01237-0.01943) | 0.01925 (0.01269-0.02654) | 0.397 |
| inertia_GLCM10_ | 8.639 (7.618-11.090) | 8.305 (4.738-12.864) | 0.475 |
| inertia_GLCM11_ | 7.899 (6.252-10.551) | 7.490 (4.903-10.988) | 0.588 |
| inertia_GLCM12_ | 10.657 (7.361-12.856) | 8.416 (5.841-13.391) | 0.373 |
| inertia_GLCM13_ | 5.349 (4.517-6.264) | 4.093 (2.640-6.714) | 0.169 |
| variance_GLCM10_ | 8.142 (5.414-9.044) | 6.598 (4.403-8.095) | 0.328 |
| variance_GLCM11_ | 8.274 (5.552-9.111) | 6.577 (4.450-8.149) | 0.328 |
| variance_GLCM12_ | 8.198 (5.407-9.130) | 6.525 (4.469-8.148) | 0.307 |
| variance_GLCM13_ | 8.259 (5.321-9.158) | 6.572 (4.477-8.225) | 0.307 |

Table 3: Interobserver agreement of PET texture features

| Parameter | Intra-class correlation coefficients |
| --- | --- |
| SUVmax | 0.936 |
| MTV | 0.931 |
| PET texture features |  |
| mean | 0.898 |
| SD | 0.974 |
| max frequency | 0.864 |
| mode | 0.435 |
| minimum | 0.425 |
| maximum | 0.988 |
| 5^th^ percentile | 0.523 |
| 10^th^ percentile | 0.555 |
| 25^th^ percentile | 0.669 |
| 50^th^ percentile | 0.807 |
| 75^th^ percentile | 0.934 |
| 90^th^ percentile | 0.976 |
| skewness | 0.515 |
| kurtosis | 0.430 |
| entropy | 0.864 |
| volume | 0.898 |
| max diameter | 0.889 |
| entropy_GLCM10_ | 0.107 |
| entropy_GLCM11_ | 0.452 |
| entropy_GLCM12_ | 0.115 |
| entropy_GLCM13_ | 0.451 |
| energy_GLCM10_ | 0.074 |
| energy_GLCM11_ | 0.297 |
| energy_GLCM12_ | 0.379 |
| energy_GLCM13_ | 0.197 |
| inertia_GLCM10_ | 0.715 |
| inertia_GLCM11_ | 0.844 |
| inertia_GLCM12_ | 0.832 |
| inertia_GLCM13_ | 0.909 |
| variance_GLCM10_ | 0.871 |
| variance_GLCM11_ | 0.906 |
| variance_GLCM12_ | 0.890 |
| variance_GLCM13_ | 0.895 |
| CT texture features |  |
| mean (HU) | 0.780 |
| SD | 0.574 |
| max-frequency | 0.847 |
| mode (HU) | 0.789 |
| minimum (HU) | 0.269 |
| maximum (HU) | 0.711 |
| skewness | 0.252 |
| kurtosis | 0.127 |
| entropy | 0.669 |
| max-diameter | 0.873 |
| entropy_GLCM10_ | 0.793 |
| entropy_GLCM11_ | 0.774 |
| entropy_GLCM12_ | 0.770 |
| entropy_GLCM13_ | 0.829 |
| energy_GLCM10_ | 0.722 |
| energy_GLCM11_ | 0.652 |
| energy_GLCM12_ | 0.682 |
| energy_GLCM13_ | 0.732 |
| inertia_GLCM10_ | 0.891 |
| inertia_GLCM11_ | 0.880 |
| inertia_GLCM12_ | 0.874 |
| inertia_GLCM13_ | 0.944 |
| variance_GLCM10_ | 0.795 |
| variance_GLCM11_ | 0.774 |
| variance_GLCM12_ | 0.776 |
| variance_GLCM13_ | 0.783 |
